# No Evidence of Sexual Dimorphism in Third Molar Agenesis Patterns: A Cross-Sectional Study of Orthodontic Patients

**DOI:** 10.3390/dj14100631

**Published:** 2026-10-02

**Authors:** Nikola Sophie Ortner, Philipp Albert Schnegelsberg, Peter Proff, Svenja Beisel-Memmert, Erika Calvano Küchler, Christian Kirschneck

**Affiliations:** 1 Department of Orthodontics, Medical Faculty, University Hospital Bonn, Welschnonnenstr. 17, 53111 Bonn, Germany; 2 Department of Orthodontics, Medical Faculty, University of Regensburg, Franz-Josef-Strauß-Allee 11, 93053 Regensburg, Germany

**Keywords:** tooth agenesis, third molars, sexual dimorphism, tooth agenesis pattern, orthodontic patients

## Abstract

**Background/Objectives**: Sexual dimorphism has been reported in several dental traits; however, its relevance in third molar agenesis (TMA) remains unclear. **Methods**: A cross-sectional retrospective study evaluated panoramic radiographs of German orthodontic patients. TMA was diagnosed using panoramic radiographs by evaluating the presence or absence of the third molars in all four quadrants of the jaws. Absence of third molar development was classified as agenesis. To ensure accuracy, diagnosis was established only in patients older than 12 years. The control group included only individuals with 32 teeth. Chi-square test was used to compare prevalence among groups. The number of TMA did not meet the assumption of normality according to the Shapiro–Wilk test, so group comparisons were performed using the Mann–Whitney U test (alpha  =  5%). **Results**: A total of 484 individuals were included (244 females, 240 males). TMA was present in 117 patients (59 females, 58 males). No sexual dimorphism or sex-specific differences in laterality were observed (*p* > 0.05). In total, 50 patients had one missing third molar, 35 patients had two missing third molars, 12 patients had three missing third molars, and 20 patients had four missing third molars. The mean number of missing third molars was 1.97 (standard error 0.16) in females and 1.88 (standard error 0.13) in males, with no significant difference between sexes (*p* = 0.69). **Conclusions**: TMA presents no sexual dimorphism as observed in other types of dental agenesis. These findings suggest that sex is unlikely to be a major factor in TMA.

## 1. Introduction

Tooth agenesis, defined as the congenital absence of one or more teeth, represents the most common developmental alteration of the human craniofacial complex [1,2]. Tooth agenesis can affect both the primary and permanent dentitions, although it occurs more frequently in the permanent dentition and most commonly affects the third molars [3]. A systematic review showed that the worldwide prevalence of TMA has been estimated at approximately 22.6%, even with substantial variation across studies and populations [1]. In contrast, agenesis of permanent teeth other than third molars is less frequent, affecting about 6.4% of the general population [2]. The phenotypic expression of tooth agenesis is highly variable, ranging from the absence of a single tooth to multiple missing teeth, and differs considerably among individuals, populations, jaws, and sexes [4,5].

Tooth agenesis may present as either syndromic or non-syndromic form. Non-syndromic tooth agenesis is more common and typically involves a limited number of teeth, while syndromic tooth agenesis is part of a broader multi-organ syndrome [6]. The aetiology of non-syndromic tooth agenesis is multifactorial, involving complex interactions between genetic and environmental factors, which likely underlie the observed differences in prevalence and patterns across populations [6].

Third molars, the teeth most commonly affected by tooth agenesis, exhibit the greatest developmental variability of all teeth [1,2]. As the last teeth to initiate and complete development, they show pronounced instability in both timing and developmental integrity, making them particularly susceptible to agenesis [1,7,8]. A meta-analysis reported that females were approximately 14% more likely than males to exhibit agenesis of at least one third molar, with maxillary TMA being more prevalent than mandibular agenesis and substantial geographic variation observed [1]. However, this meta-analysis treated TMA as a binary outcome and did not assess sexual dimorphism in specific agenesis patterns or in relation to the absence of other permanent teeth [9]. Importantly, previous studies have primarily focused on prevalence, whereas pattern distribution, laterality, and severity of TMA in relation to sex remain insufficiently characterized.

While sex-specific effects in dental agenesis have been repeatedly reported, sex dimorphism in TMA patterns and severity has rarely been studied. In particular, the relationship between the agenesis of teeth other than third molars in men and women remains unclear. Prior findings link the number of teeth formed to the size and shape of the facial skull. Individuals with tooth agenesis tend to have smaller facial contours, even in cases where agenesis is limited to third molars. Interestingly, these associations do not appear to be significantly mediated by sex [10,11,12].

Whether TMA exhibits sexual dimorphism remains an open question. Answering it could improve clinical risk assessment and diagnostic accuracy, and inform our understanding of the biological mechanisms linking tooth formation, craniofacial development, and human variation [13].

The aim of the present study was to investigate sexual dimorphism in the patterns and severity of TMA in orthodontic patients.

## 2. Materials and Methods

### 2.1. Design, Study Setting and Ethics

This retrospective cross-sectional study was undertaken to investigate the prevalence, patterns, and potential sex-specific differences in TMA within a group of German orthodontic patients. The dependent variable was third molar agenesis, and the independent variable was sex, which was self-reported during the initial dental anamnesis. The participants were consecutively selected.

For the presentation of this study, the STROBE (strengthening the reporting of observational studies in epidemiology) statement was followed as a guideline [14].

This research project was conducted at the Department of Orthodontics, University Hospital Bonn (2024-252-BO) and University Hospital Regensburg (19-1549-101), Germany. Patient records and panoramic radiographs obtained as part of routine orthodontic diagnostics between 3 September 2012 and 22 June 2020 (for the sample from Regensburg) and between 30 April 2009 and 11 February 2025 (for the sample from Bonn) were retrospectively reviewed. Each radiograph was systematically examined for the presence of tooth germs, crowns, or other detectable developmental stages of the third molars between 20 May 2020 and 1 February 2021 (for the sample from Regensburg) and between 17 June 2024 and 22 August 2025 (for the sample from Bonn).

### 2.2. Study Selection

To ensure diagnostic validity and minimize the risk of misclassification due to ongoing dental development, only patients who had at least one panoramic radiograph (baseline or follow-up) taken at age 12 years or older were included, as third molar initial development is generally detectable by this age. All radiographic assessments were conducted under standardized conditions, with careful attention to imaging quality and anatomical landmarks, to maintain consistency and reproducibility across the study sample. In order to minimize potential bias and ensure data integrity, individuals presenting with syndromic conditions, cleft lip and/or palate or an unverified history of previous extraction of third molars were excluded from the analysis. Because early-stage patients could be monitored longitudinally for third molar germ development, those initiating treatment at an older age with ambiguous third molar status—where prior extractions could not be confirmed or ruled out—were excluded. For the purposes of analysis, the study population was stratified into two distinct groups. The study group included patients exhibiting agenesis of one or more third molars, whereas the control group comprised individuals with a complete permanent dentition, including all 32 teeth. Individuals presenting with agenesis of teeth other than third molars were excluded from the control group to ensure that the control population was free of any dental agenesis and to minimize potential confounding effects associated with other forms of tooth agenesis.

### 2.3. Panoramic Radiograph Analysis and Data Collection

Panoramic radiographs were selected as a diagnostic tool due to their comprehensive visualization of the entire dentition, including all four jaw quadrants, allowing for reliable assessment of third molar development [15]. Digital panoramic radiographs were examined in a room with controlled ambient light using dedicated high-resolution displays.

All panoramic radiographs were examined using the same protocol. In case of doubt, more than one panoramic radiograph of the patient was evaluated to confirm TMA diagnosis. Panoramic radiographs were evaluated by one experienced calibrated dentist as previously reported [16].

Each quadrant was systematically examined for the presence of third molar tooth germs; agenesis was recorded when no signs of developmental crypt formation or calcification were visible. In cases where no evidence of third molar formation was detected within the anatomically expected regions, the tooth was classified as congenitally absent.

### 2.4. Sample Size Determination

The sample size was determined with reference to previously published studies investigating TMA and sexual dimorphism [9,17]. The final sample size was therefore based on sample sizes commonly reported in prior studies in this field, and the differences observed between males and females. Sample size calculation was based on detecting a 10% difference in prevalence between sexes (α = 0.05, β = 0.80), resulting in a required sample of approximately 480 individuals.

### 2.5. Statistical Analysis

Data were compiled and managed in a spreadsheet using Microsoft Excel (Microsoft Corporation, Redmond, WA, USA). All statistical analyses were performed using GraphPad Prism for Windows, Version 11.0.0. The prevalence of TMA between groups was compared using the chi-square test to assess potential differences in categorical variables. Prior to comparing the number of missing third molars between groups, data distribution was evaluated for normality using the Shapiro–Wilk test. As the assumption of normality was not met, intergroup comparisons were conducted using the non-parametric Mann–Whitney U test. All statistical tests were two-tailed, and the level of significance was set at α = 0.05. Data are presented as mean ± standard error of the mean (SEM), where applicable.

Potential confounding was addressed through predefined eligibility criteria, thereby minimizing heterogeneity within the study population. No additional multivariable adjustment was performed, as the study population was relatively homogeneous with respect to clinical indication (orthodontic patients). However, potential residual confounding cannot be excluded.

Subgroup analyses were conducted to explore differences according to sex. There was no missing data. Cases with incomplete or non-evaluable records were excluded from the respective analyses (complete-case analysis). No data imputation procedures were applied.

Given the retrospective design of this cross-sectional study, loss to follow-up was not applicable. The analytical approach accounted for the defined sampling framework by applying uniform inclusion and exclusion criteria across all participants.

No formal sensitivity analyses were performed.

## 3. Results

### 3.1. Study Selection and Characteristics

**Figure 1:**
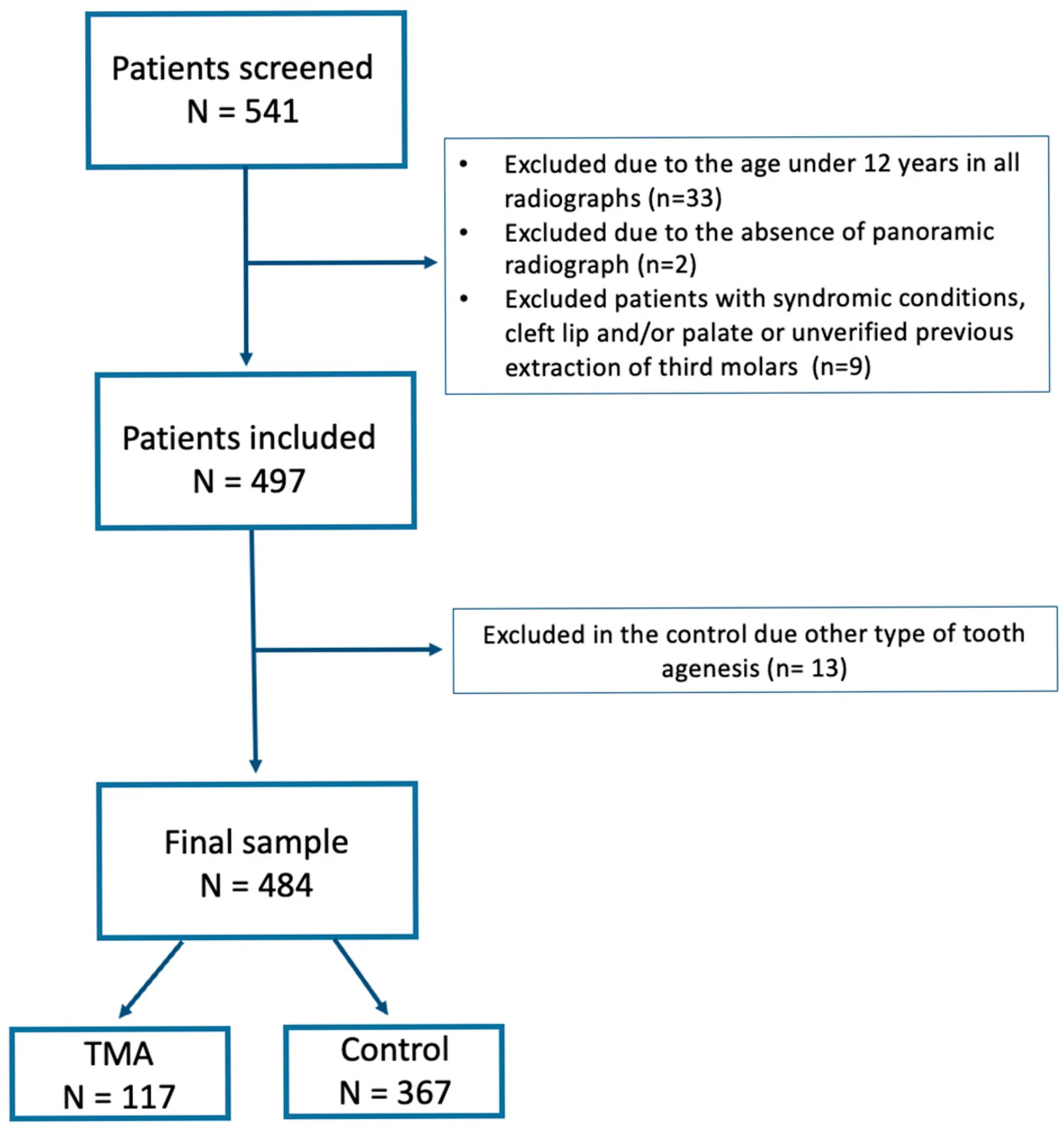
Flowchart. Notes: TMA means Third Molar Agenesis.

**Figure 2:**
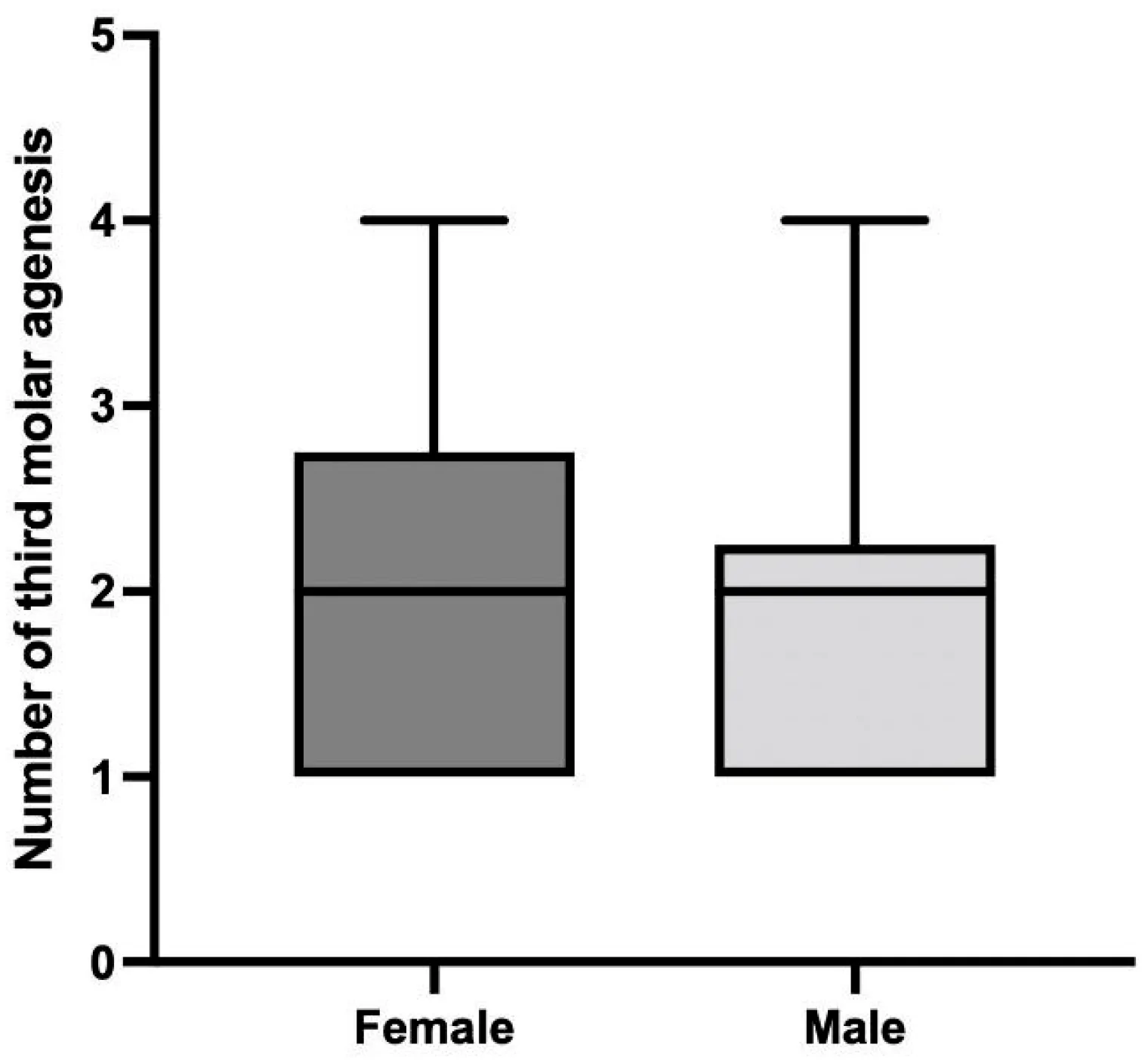
Number of third molar agenesis distribution according to the sex. Mann–Whitney U test was used. Notes: TMA means Third Molar Agenesis.

Figure 1 shows the flowchart of the study. A total of 484 individuals were included in the study, comprising 244 females (50.4%) and 240 males (49.6%). As illustrated in Figure 2, the distribution of the number of missing third molars was comparable between sexes across all categories.

The baseline demographic and dental characteristics of the study population are summarized in Table 1. Among affected individuals, 59 were females and 58 were males, demonstrating a nearly equal distribution across sexes. Statistical analysis revealed no significant sex-specific differences in the prevalence of TMA (*p* = 0.99). Similarly, the laterality of missing third molars (unilateral versus bilateral) did not differ significantly between women and men (Table 1).

### 3.2. Distribution According to the Sex

Table 2 shows the TMA prevalence and third molar subtype prevalence according to the sex. The number of missing third molars per individual varied from one to four. Specifically, 50 patients exhibited agenesis of a single third molar, 35 patients had two missing third molars, 12 patients had three missing third molars, and 20 patients were missing all four third molars. Figure 2 shows the comparison between females and males with TMA. The mean number of missing third molars was 1.97 (standard error [SE] 0.16) in women and 1.88 (SE 0.13) in men, with no significant difference between the sexes (*p* = 0.69). The observed differences were small and are unlikely to be clinically meaningful.

Further analysis of third molar subtypes revealed a relatively uniform distribution of agenesis patterns across the maxilla and mandible, with no significant sex-specific differences. Odds ratios close to 1 further support the absence of clinically relevant sex-specific effects (Table 2). In addition, cases of TMA associated with other dental agenesis were observed in both sexes, but the frequency of such combined agenesis did not differ significantly between females and males.

Table 3 provides the distribution patterns of TMA according to the laterality. Unilateral and bilateral agenesis patterns were similarly distributed in both sexes.

## 4. Discussion

Sexual dimorphism appeared to play no role in TMA in our sample, whether in terms of prevalence, severity, or laterality. While most previous studies on TMA focused on prevalence alone, the present study additionally assessed symmetry and severity patterns, allowing a more comprehensive evaluation of potential sex-specific effects than has typically been reported.

Although the literature indicates that tooth agenesis exhibits sexual dimorphism [17,18,19], sex-associated differences in TMA have rarely been examined. Our findings are consistent with previous studies showing little to no evidence of sexual dimorphism in TMA. Studies in both orthodontic samples and population-based samples similarly found no clinically relevant sex-specific differences [9,10,11,18,20]. Only minor variations between males and females have been reported without statistical significance. By additionally analyzing symmetry and severity, the present study provides a more comprehensive assessment of potential sexual dimorphism. The results of the current study were mostly consistent across the evaluated parameters, indicating that TMA affected males and females similarly. Nevertheless, bilateral TMA was observed slightly more often in females compared to males, whereas TMA associated with additional tooth agenesis occurred slightly more frequently in males. These differences did not reach statistical significance and should be interpreted with caution. It is therefore possible that a larger and more diverse study population would be required to detect a true underlying sex-specific effect. Population-specific characteristics, such as geographic or ethnic background, may contribute to the discrepancies observed between individual studies and broader meta-analytic findings.

While individual studies, including ours, have largely failed to detect sex-specific differences, broader meta-analytic evidence has suggested a slightly higher overall prevalence of TMA in females. Carter and Worthington (2015) reported a female predominance in their systematic review, but noted considerable variation in the underlying study-differences in geographic region, ethnic background, age range, and diagnostic methodology among them [1]. This variation may explain why sexual dimorphism observed in the meta-analysis is not consistently seen across individual studies and may also suggest that the observed differences are related to population-specific characteristics or differences in study design.

TMA also appears to follow different patterns than the agenesis of other permanent teeth. Nonsyndromic tooth agenesis excluding third molars has repeatedly been shown to exhibit sexual dimorphism in both prevalence and distribution [17]. Certain teeth, such as maxillary lateral incisors or premolars, have been reported to be more frequently absent in one sex, depending on the population studied [17]. Kanchanasevee et al. (2023) further demonstrated sexual dimorphism in the severity of agenesis, with single-tooth agenesis occurring more frequently in males, whereas agenesis affecting two or more teeth was more prevalent in females [17]. These findings demonstrate that sexual dimorphism exists for teeth other than third molars, affecting both overall prevalence and severity, with females generally showing higher agenesis rates. This suggests that a biological mechanism underlying sexual dimorphism in tooth development exists in principle, but that third molars appear to be less strongly affected. The slightly higher prevalence of bilateral TMA among females observed in our study indicates that sexual dimorphism could also be present in TMA, although it may be less pronounced than in other teeth.

Third molars are among the most variable teeth in the human dentition, particularly regarding their development and agenesis patterns. Their late development, prolonged developmental period and evolutionary adaptability may explain this unique biological characteristic. At the molecular level, odontogenesis is regulated by complex genetic and epigenetic networks, including signaling pathways involving MSX1, PAX9, AXIN2, and components of the WNT pathway [21,22,23]. While these pathways have been implicated in tooth agenesis more broadly, there is currently no compelling evidence that they exert strong sex-dependent effects specifically on third molar development.

Alamoudi et al. (2022) proposed that TMA is under substantial genetic, and potentially epigenetic control, affecting males and females in a largely comparable manner [9].

From an evolutionary perspective, the progressive reduction in jaw size and tooth number in modern humans has been interpreted as part of an ongoing microevolutionary process [11,12]. As the terminal elements of the dental arch and the last teeth to develop, third molars may be particularly susceptible to such evolutionary pressures [5]. The absence of detectable sexual dimorphism in this context could indicate that evolutionary forces influencing third molar reduction act similarly across sexes, rather than differentially affecting male and female developmental trajectories [9].

In accordance with the STROBE recommendations for observational studies, several methodological aspects merit consideration. A key strength of the present study is the relatively balanced sex distribution and the use of standardized radiographic assessment within a clearly defined orthodontic sample. Furthermore, the comprehensive evaluation of agenesis patterns, symmetry (unilateral versus bilateral), and number of missing third molars extends the analysis beyond simple prevalence estimates and enhances the interpretative robustness of the findings. Nevertheless, certain limitations should be acknowledged. The study population consisted exclusively of orthodontic patients, which may limit the generalizability of the findings to the broader German population. Orthodontic samples may exhibit an increased prevalence of dental anomalies compared with community-based samples. Another inherent limitation relates to the recruitment setting: because subtle malocclusions or midline deviations associated with asymmetrical agenesis patterns can drive patients to seek orthodontic care, our sample may reflect a degree of referral bias rather than a truly representative population-based cohort. This could influence the observed distribution of agenesis and limit the external validity and generalizability of the findings. In our study selection, individuals with isolated non-third molar agenesis were excluded from the control group to preserve a strictly non-agenesis baseline, even though TMA accompanied by other missing teeth was retained in the study group. While intended to prevent phenotype misclassification in the control sample, this asymmetric criterion may be considered an inherent limitation when interpreting the findings. Furthermore, ethnic and geographic variability in the prevalence and patterns of tooth agenesis may affect the applicability of the findings to other populations. The cross-sectional design does not allow causal conclusions to be drawn regarding developmental mechanisms or temporal associations. Although the sample size was substantial, very small differences between sexes cannot be definitively excluded, and minor effects may remain undetected. Although no significant differences were detected, the study may not be powered to detect minor effects. Finally, no multivariable analysis was performed, limiting the ability to adjust for potential confounding factors and to evaluate the independent effect of sex on third molar agenesis. Potential residual confounding factors, including intra-population ethnic variability and environmental influences on dental development, were not stratified in the present analysis. These factors may also contribute to variation in agenesis prevalence across different contexts and should be addressed in future investigations. Further studies would benefit from large, multicenter and more diverse samples using standardized diagnostic criteria to reduce methodological heterogeneity across studies. Multivariable analyses and consideration of population-specific factors, including ethnic and geographic variability, may help clarify whether observed differences reflect true biological variation or cohort-specific characteristics. Investigating sexual dimorphism in TMA across patient groups beyond the orthodontic setting may help clarify whether the absence of sex-related effects observed in this study is specific to orthodontic populations. Finally, sex-specific effects cannot be completely excluded.

## 5. Conclusions

In conclusion, this study found no evidence of sexual dimorphism in TMA in a group of German orthodontic patients. These findings suggest that sex is unlikely to be a clinically relevant factor in the assessment of TMA.

## Figures and Tables

**Table 1:** Sample characteristics and subtypes of third molar agenesis.

Variables	N
Sex distribution	
Females	244
Males	240
Third molar agenesis characteristics	
TMA (at least one third molar missing)	117
Maxillary third molar agenesis	77
Mandibular third molar agenesis	73
Third molar agenesis associated with another missing tooth/teeth	12
Unilateral third molar agenesis	51
Bilateral third molar agenesis	66

Notes: TMA means Third Molar Agenesis. The table shows the prevalence of third molar agenesis according to the sex.

**Table 2:** Third molar agenesis distribution according to the sex.

Groups	Females	Males	*p*-Value
All 32 teeth	185 (50.4)	182 (49.6)	reference
Third molar agenesis	59 (50.4)	58 (49.6)	0.99
Maxillary third molar agenesis	42 (54.5)	35 (45.5)	0.51
Mandibular third molar agenesis	36 (49.3)	37 (50.7)	0.86
Third molar agenesis associated with another agenesis	5 (41.7)	7 (58.3)	0.55

Note: All comparisons were performed using the group “All 32 teeth” as controls. Chi-square was used.

**Table 3:** Laterality distribution according to the sex.

Groups	Females	Males	*p*-Value	OR (CI 95%)
Unilateral third molar agenesis	23 (45.1)	28 (54.9)	0.31	1.46 (0.67–2.98)
Bilateral third molar agenesis	36 (54.5)	30 (45.5)		

Note: OR means odds ratio, CI means confidence interval. Chi-square was used.

## Data Availability

The data presented in this study are not publicly available due to a patient privacy restriction but may be available from the corresponding author upon reasonable request.

## Abbreviations

The following abbreviations are used in this manuscript:

TMA	Third Molar Agenesis
OR	Odds Ratio
CI	Confidence Interval
